# Prevalence of Depression among University Students: A Systematic Review and Meta-Analysis Study

**DOI:** 10.1155/2013/373857

**Published:** 2013-09-25

**Authors:** Diana Sarokhani, Ali Delpisheh, Yousef Veisani, Mohamad Taher Sarokhani, Rohollah Esmaeli Manesh, Kourosh Sayehmiri

**Affiliations:** ^1^Student Research Committee, Ilam University of Medical Science, P.O. Box 69311-57793, Ilam, Iran; ^2^Department of Computer, Faculty of Engineering, Malayer University, P.O. Box 95863-65719, Hamadan, Iran; ^3^Department of Clinical Epidemiology, Ilam University of Medical Sciences, P.O. Box 69315-138, Ilam, Iran; ^4^Psychosocial Injuries Research Center, Ilam University of Medical Science, P.O. Box 69315-138, Ilam, Iran; ^5^Science and Research Branch, Islamic Azad University, Kermanshah, Iran; ^6^Eslamabad Payame Noor University, Kermanshah, Iran; ^7^Social Medicine Department, Medicine Faculty, Ilam University of Medical Sciences, P.O. Box 69315-138, Ilam, Iran

## Abstract

*Introduction*. Depression is one of the four major diseases in the world and is the most common cause of disability from diseases. The aim of this study is to estimate the prevalence of depression among Iranian university students using meta-analysis method. *Materials and Methods*. Keyword depression was searched in electronic databases such as PubMed, Scopus, MAGIran, Medlib, and SID. Data was analyzed using meta-analysis (random-effects model). Heterogeneity of studies was assessed using the *I* ^2^ index. Data was analyzed using STATA software Ver.10. *Results*. In 35 studies conducted in Iran from 1995 to 2012 with sample size of 9743, prevalence of depression in the university students was estimated to be 33% (95% CI: 32–34). The prevalence of depression among boys was estimated to be 28% (95% CI: 26–30), among girls 23% (95% CI: 22–24), single students 39% (95% CI: 37–41), and married students 20% (95% CI: 17–24). Metaregression model showed that the trend of depression among Iranian students was flat. *Conclusions*. On the whole, depression is common in university students with no preponderance between males and females and in single students is higher than married ones.

## 1. Introduction

Depression among university students is extremely prevalent and widespread problem across the country [1–3]. University students are a special group of people that are enduring a critical transitory period in which they are going from adolescence to adulthood and can be one of the most stressful times in a person's life. Trying to fit in, maintain good grades, plan for the future, and be away from home often causes anxiety for a lot of students [4]. As a reaction to this stress, some students get depressed. They find that they cannot get themselves together. They may cry all of the time, skip classes, or isolate themselves without realizing they are depressed. Previous studies reported that depression in university students is noted around the world [5–7] and the prevalence seems to be increasing [8].

The average age of onset is also on the decline, making depression a particularly salient problem area for university student populations [8]. Over two-thirds of young people do not talk about or seek help for mental health problems [9]. 

In Iran, preliminary studies on emotional distress have emerged in recent years including depression in Iranian university. Within the abovementioned background, the aim of this study is to estimate the prevalence of depression among university students using meta-analysis method.

## 2. Methods and Materials

### 2.1. Literature Search

Our search strategy, selection of publications, and the reporting of results for the review will be conducted in accordance with the PRISMA guidelines [10]. Literatures on the depression among student were acquired through searching Scientific Information Databases (SID), Global Medical Article Limberly (Medlib), Iranian Biomedical Journal (Iran Medex), Iranian Journal Database (Magiran), and international databases including PubMed/Medline, Scopus and ISI Web of Knowledge. The search strategy was limited to the Persian and/or English language and articles published up until February 2012 were considered. All publications with medical subject headings (MeSh) and keywords in title, abstract, and text for words including student depression were investigated. Iranian scientific databases were searched only using the keyword “student depression,” as these databases do not distinguish synonyms from each other and do not allow sensitive search operation using linking terms such as “AND,” “OR” or “NOT.” Consequently, this single keyword search was the most practical option.

### 2.2. Selection and Quality Assessment of Articles

All identified papers were critically appraised independently by two reviewers. Disagreements between reviewers were resolved by consensus. Appraisal was guided by a checklist assessing clarity of aims and research questions. The inclusion criteria were as follows: (1) studies in the mentioned databases with full text, despite the language of original text; (2) having a standardized assessment of depression (either self-report or observer-rated). Exclusion criteria were (1) studies upon student overlapping time intervals of sample collection from the same origin; (2) low-quality design (STROBE checklist score's below 7.75 [11]); (3) inadequate reporting of results.

### 2.3. Data Extraction

Data were extracted using a standardized and prepiloted data extraction form. Data extraction will be undertaken by the first reviewer, and checked by a second reviewer although the process will be discussed and piloted by both reviewers. All identified papers will be critically appraised independently by both reviewers. Disagreements were resolved through discussion. Appraisal will be guided by a checklist assessing clarity of aims and research questions. Information was extracted from each included study (including author, title, year and setting of study, methods of sample selection, sample size, study type, age, STROBE score, and prevalence). These data abstraction forms were reviewed and eligible papers were entered into the meta-analysis.

### 2.4. Statistical Analysis

The random effects model was used for combining results of studies in meta-analysis. Variance for each study was calculated using the binomial distribution formula. The presence of heterogeneity was determined by the DerSimonian-Laird (DL) approach [12]. Significance level was <0.1 and *I* ^2^ statistic for estimates of inconsistency between studies. The *I* ^2^ statistic estimates the percent of observed between-study variability due to heterogeneity rather than to chance and ranges from 0 to 100 percent (values of 25%, 50% and 75% were considered representing low, medium and high heterogeneity, resp.). A value of 0% indicates no observed heterogeneity whilst 100% indicates significant heterogeneity. For this review, we determined that *I* ^2^ values above 75 percent were indicative of significant heterogeneity warranting analysis with a random effects model as opposed to the fixed effects model to adjust for the observed variability [13]. This heterogeneity was further explored through subgroup analyses and metaregression. Univariate and multivariate approach were employed to assess the causes of heterogeneity among the selected studies. Egger test was conducted to examine potential publication bias. Data manipulation and statistical analyses were done using STATA software, version 10. *P* values < 0.05 were considered as statistically significant.

## 3. Results 

**Figure 1:**
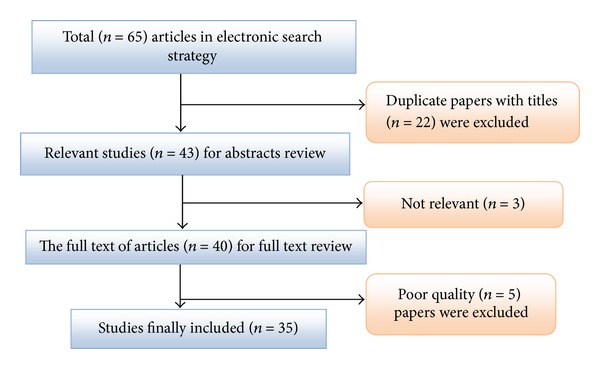
Results of the systematic literature search.

According to the literature search strategies, 65 studies were identified, but 30 studies were excluded as they did not meet the inclusion criteria. Finally, 35 studies were published between 1995 and 2012 and included in meta-analysis (Table 1 and Figure 1).

**Figure 2:**
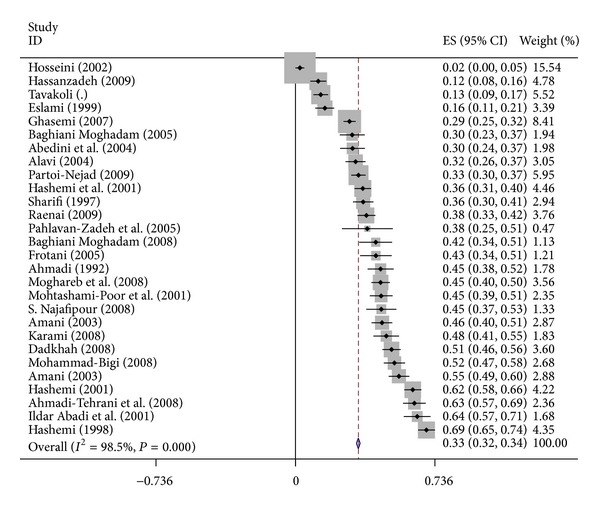
Forest plots of student depression for random effects meta-analyses. (Squares represent effect estimates of individual studies with their 95% confidence intervals of depression with size of squares proportional to the weight assigned to the study in the meta-analysis. The diamond represents the overall result and 95% confidence interval of the random-effects meta-analysis.)

**Figure 3:**
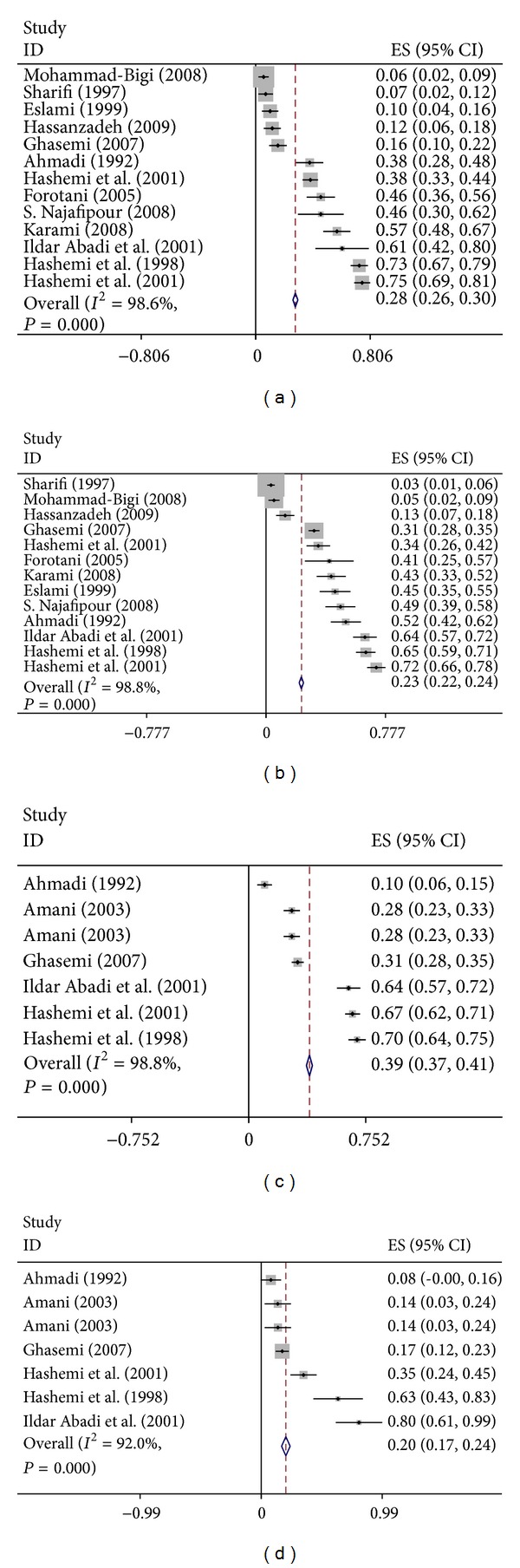
Forest plots of student depression for subgroups analysis (forest plot (a) depression among male students, (b) among female students, (c) among single students, and (d) among married students).

The overall prevalence of depression among university students was 33% (CI 95%: 32–34) (Figure 2). Prevalence of depression among subgroup including male and female students and single and married students was 28% (CI 95%: 26–30), 23% (CI 95%: 22–24), 39% (95%: 37–41), and 20% (CI 95%: 17–24) respectively (Figure 3).

**Figure 4:**
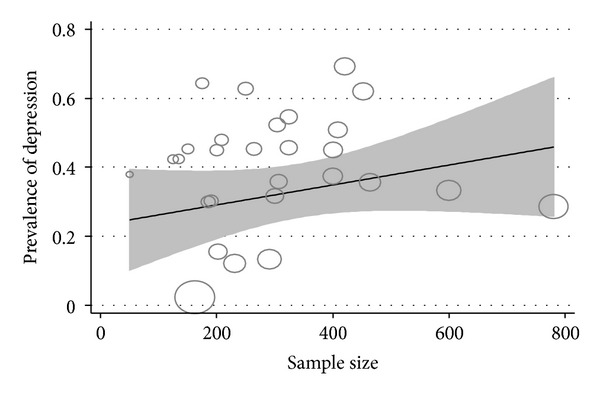
Meta-regression plots of change in depression according to changes in continuous study moderator's sample size.

**Figure 5:**
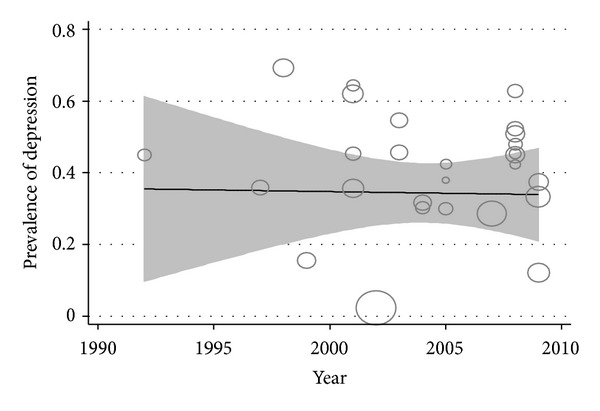
Meta-regression plots of change in depression according to changes in continuous study moderator's year.

The meta regression of the prevalence of student depression again sample size of studies showed no statistically significant relationship (*P* = 0.66) (Figure 4). Scatter plot year of study and the prevalence of student depression meta regression showed a negative and no statistically significant relationship (*P* = 0.70). Since 1995, the student depression showed a stable trend (Figure 5).

## 4. Discussion 

In this systematic review, we have fully described our search strategy, study selection, data summary, and analysis to allow sensitivity analysis of any aspect of our approach. We have included every study that to our knowledge satisfies our inclusion criteria and employed techniques of estimation that allow integration of studies with high heterogeneity. In situations with high between-study heterogeneity (93.3%), the use of random-effects models is recommended as it produces study weights that primarily reflect the between-study variation and thus provides close-to-equal weighting [13].

In the current study, the Beck depression inventory (BDI) has been utilized to detect the prevalence of depression among university students. Although it is not designed for diagnostic purposes, its epidemiologic utility has been evaluated in several studies, which concluded that it is a reliable and valid instrument for detecting depressive disorders in nonclinical populations. Several studies support the BDI's usefulness in measuring and predicting depression in adolescent samples [46, 47].

The study showed that the prevalence of depression among university student was 33% (CI 95%: 32–34). Steptoe et al. showed that Asian countries had the highest level depressive symptoms [48], which was consistent with our result. The incidence of depression in our study was higher than in other studies, and as Bayram and Bilgel reported that depression were found in 27.1% of Turkish university students [49], Bostanci reported that out of all university students in Denizli, 26.2% had a BDI score of 17 or higher [50]. This variation has been explained to be due to cultural differences, different measurement tools, different methods, and different appraisal standards. University is an important transient life stage, with special academic, financial, and interpersonal pressures. Undergoing these transitions may lead to an increased risk of depression. However, the prevalence of depressive symptoms in the present study is a high incidence rate, more than that seen in average people. Most students who join university in Iran are leaving their homes for the first time. This might subject them to loss of the traditional social support and supervision, in addition to residing with other students and peer relationships. Moreover, there is a change in the style of learning from what the students are used to in school. These changes may act as risk factors to depression in university students in Iran.

We found no differences in depression between genders in our study. Similar to our results, some previous studies [49–51] showed that no differences in depression were observed among male and female students. This might originate from the fact that Iranian female university students have equal experience of the same pressure. However, some studies findings are contrary to our results and found higher levels of depression among female students [50–52]. 

We found that single students were susceptible to depression compared with married students. This may be because the single students face more stressful events than the married students, such as employment, economic, graduation, and marriage pressures. Contrary to our study, some studies showed that married students reported higher levels of depression [49]. 

One of the limitations of this study is that the difference in assessment tools and researchers varies in their choice of cut point according to the study location. Secondly, the more studies were observational and patients were not randomly chosen in addition our ability to assess study quality was limited by the fact that many studies failed to offer detailed information on selected subjects or valid data on important factors. Therefore selection bias and confounding seem inevitable. Thirdly, many of our data were extracted from the internal databases in Iran.

## 5. Conclusion 

In summary, we found that depression is common in university students with no preponderance between males and females and in single students is higher than married ones. Our findings point to importance of screening of this vulnerable population and taking appropriate interventional measures to prevent the complications of depression. Further research studying sociodemographic factors and the effect of depression on the academic performance is needed. 

## Figures and Tables

**Table 1:** Feature and characteristic studies included in study.

Study number/author(s)/no. of reference	Place	Publication year	No. of population	Prevalence (%)	Instrument assessment	Cut point
(1) Bahrami Dashtaki [14]	Tehran	2005	100	—	BDI	15
(2) Mohammadian [15]	Tehran	2010	302	—	BDI	16
(3) Alavi [16]	Mashhad	2011	20	—	BDI	16
(4) Hosseini [17]	Kermanshah	2002	162	23.5	BDI	15
(5) Bahadori Khosroshahi [18]	Zahedan	2010	200	—	BDI	16
(6) Biani [19]	Tabriz	2008	571	—	BDI	16
(7) Mohammad-Bigi et al. [20]	Arak	2009	304	52.3	BDI	15
(8) Amani et al. [21]	Ardabil	2004	324	54.7	BDI	16
(9) Dadkhah [22]	Ardabil	2009	409	50.8	BDI	16
(10) Pahlavan-Zadeh et al. [23]	Isfahan	2010	50	38	GHQ 28	22
(11) Ranjbar-Kohan and Sajjadi Nejad [24]	Isfahan	2010	40	—	BDI	16
(12) Makvandi et al. [25]	Ahvaz	2012	185	—	BDI	17
(13) Makvandi [26]	Ahvaz	2010	215	—	BDI	16
(14) Ahmadi [27]	Ahvaz	1995	200	45	BDI	16
(15) Hasan Zadeh Taheri et al. [28]	Birjand	2011	231	12.1	BDI	14
(16) Moghareb et al. [29]	Birjand	2009	400	45	BDI	16
(17) Frotani [3]	Lar	2005	134	42.5	BDI	16
(18) Najafipour and Yektatalab [30]	Jahrom	2008	150	45.4	BDI	15
(19) Ildar Abadi et al. [1]	Zabol	2002	175	64.3	BDI	16
(20) Ahmadi-Tehrani et al. [31]	Qom	2009	250	62.8	BDI	14
(21) Partoi-Nejad [32]	Qom	2011	600	33.3	GHQ 28	22
(22) Karami [33]	Kashan	2009	208	48	GHQ 28	22
(23) Sooky et al. [34]	Kashan	2010	307	35.8	BDI	16
(24) Raenai et al. [35]	Kordestan	2010	400	37.5	BDI	17
(25) Eslami et al. [36]	Gorgan	2002	202	15.5	BDI	16
(26) Abdollahi et al. [37]	Golestan	2011	132	—	BDI	16
(27) Tavakoli et al. [38]	Gonabad	2001	291	13.4	BDI	15
(28) Ghasemi et al. [39]	Mashhad	2009	780	28.6	BDI	15
(29) Mohtashami-Poor et al. [40]	Mashhad	2001	264	45.3	BDI	16
(30) Abedini et al. [2]	Bandaradas	2007	190	30.2	BDI	16
(31) Hashemi et al. [41]	Yasuj	2003	421	69.2	BDI	16
(32) Hashemi et al. [42]	Hormozgan	2004	452	62	BDI	14
(33) Hashemi and Kamkar [43]	Yasuj	2001	464	35.6	BDI	17
(34) Baghiani Moghadam and Ehrampoosh [44]	Yazd	2006	125	42.4	BDI	16
(35) Baghiani Moghadam et al. [45]	Yazd	2011	185	30	BDI	15
